# Influence of Intraoperative Hemodynamic Parameters on Outcome in Simultaneous Pancreas–Kidney Transplant Recipients

**DOI:** 10.3390/jcm11071966

**Published:** 2022-04-01

**Authors:** Robert Sucher, Tina Schiemanck, Hans Michael Hau, Sven Laudi, Sebastian Stehr, Elisabeth Sucher, Sebastian Rademacher, Daniel Seehofer, Nora Jahn

**Affiliations:** 1 Department of Visceral, Transplantation, Vascular and Thoracic Surgery, University Hospital of Leipzig, 04103 Leipzig, Germany; robert.sucher@medizin.uni-leipzig.de (R.S.); tina-schiemanck@outlook.de (T.S.); hans-michael.hau@uniklinikum-dresden.de (H.M.H.); sebastian.rademacher@medizin.uni-leipzig.de (S.R.); daniel.seehofer@medizin.uni-leipzig.de (D.S.); 2 Department of Anesthesiology and Intensive Care Medicine, University Hospital of Leipzig, 04103 Leipzig, Germany; sven.laudi@medizin.uni-leipzig.de (S.L.); sebastian.stehr@medizin.uni-leipzig.de (S.S.); 3 Department of Visceral, Thoracic and Vascular Surgery, Faculty of Medicine Carl Gustav Carus, University Hospital, Technische Universität Dresden, 01307 Dresden, Germany; 4 Department of Oncology, Gastroenterology, Hepatology, Pneumology and Infectiology, University Hospital of Leipzig, 04103 Leipzig, Germany; elisabeth.sucher@medizin.uni-leipzig.de

**Keywords:** simultaneous pancreas–kidney transplantation, graft outcome, patient outcome, mean arterial pressure, hemodynamic monitoring

## Abstract

Objectives: Adequate organ perfusion, as well as appropriate blood pressure levels at the time of unclamping, is crucial for early and long-term graft function and outcome in simultaneous pancreas–kidney transplantation (SPKT). However, the optimal intraoperative mean arterial pressure (MAP) level has not well been defined. Methods: From a prospectively collected database, the medical data of 105 patients undergoing SPKT at our center were retrospectively analyzed. A receiver operating characteristic (ROC) analysis was preliminarily performed for optimal cut-off value for MAP at reperfusion, to predict early pancreatic graft function. Due to these results, we divided the patients according to their MAP values at reperfusion into <91 mmHg (*n* = 47 patients) and >91 mmHg (*n* = 58 patients) groups. Clinicopathological characteristics and outcomes, as well as early graft function and long-term survival, were retrospectively analyzed. Results: Donor and recipient characteristics were comparable between both groups. Rates of postoperative complications were significantly higher in the <91 mmHg group than those in the >91 mmHg group (vascular thrombosis of the pancreas: 7 (14%) versus 2 (3%); *p* = 0.03; pancreatitis/intraabdominal abscess: 10 (21%) versus 4 (7%); *p* = 0.03; renal delayed graft function (DGF): 11 (23%) versus 5 (9%); *p* = 0.03; postreperfusion urine output: 106 ± 50 mL versus 195 ± 45 mL; *p* = 0.04). There were no significant differences in intraoperative volume repletion, central venous pressure (CVP), use of vasoactive inotropic agents, and the metabolic outcome. Five-year pancreas graft survival was significantly higher in the >91 mmHg group (>91 mmHg: 82% versus <91 mmHg: 61%; *p* < 0.01). No significant differences were observed in patient and kidney graft survival at 5 years between both groups. Multivariate Cox regression analysis affirmed MAP < 91 mmHg as an independent prognostic predictor for renal DGF (HR 3.49, 1.1–10.8, *p* = 0.03) and pancreas allograft failure (HR 2.26, 1.0–4.8, *p* = 0.01). Conclusions: A MAP > 91 mmHg at the time point of reperfusion was associated with a reduced rate of postoperative complications, enhancing and recovering long-term graft function and outcome and thus increasing long-term survival in SPKT recipients.

## 1. Introduction

Simultaneous pancreas–kidney transplantation (SPKT) represents the international gold standard treatment for patients with insulin-dependent diabetes mellitus type 1 (IDDM1) and end-stage kidney disease (ESKD) [1]. Due to continuous surgical and immunosuppressive improvements within recent decades, SPKT provides significant survival benefits, as well as resulting in a better quality of life (QOL), compared with deceased donor kidney transplantation alone (KTA) [2,3,4,5]. However, despite these encouraging outcomes, SPKT remains associated with the highest risk of postoperative complications of all abdominal organ transplantations, and graft failure continues to occur in many cases as a result of various causes [4,5,6,7]. In this context, delayed graft function (DGF) and surgical-related influences play major roles in reduced graft and patient survival in this transplant type [7,8,9,10]. Well-established donor- and recipient-related specific risk factors include obesity, diabetes, severely limited cardiac function, donor age > 55 years, male gender, increased waiting time before organ transplantation, dialysis requirement prior to transplantation, and ischemia times for SPKT [8,11,12]. However, most of these outcome influencing factors are not modifiable or emendable at the time point of allocation of the donated organ to a transplantation center. However, there is still insufficient and inconclusive evidence on modifiable hemodynamic factors on short- and long-term graft outcomes in abdominal organ transplantation, particularly in the type of SPKT [12,13,14,15,16]. Specifically, maintaining adequate perfusion of newly implanted organs during the operative procedure is in fact considerably important in the development and prevention of postoperative complications [15,16]. Previous reports were able to prove that, in the case of KTA, hypoperfusion is associated with acute renal injury, vessel thrombosis, and subsequent deterioration in graft outcomes [15,16]. Mean arterial blood pressure (MAP) is one of the main intraoperative surrogate parameters for adequate organ perfusion and serves as an indirect measurement of renal perfusion in clinical practice [13,17,18]. Due to the lack of autoregulation of both, renal and pancreas transplants, keeping MAP within physiological limits seems reasonable; however, this parameter gained increased attention during the last decade [12,13,14,16,19]. There have been some studies on blood pressure management and optimized target levels of MAP at the time point of reperfusion in KTA, showing promising results; however, little is known about ideal MAP, appropriate intravascular volume status, or vasopressor administration in SPKT recipients during reperfusion [12,16,20,21].

The aim of this study was to evaluate and validate the association of peri- and intraoperative hemodynamic parameters, with a special focus on the recipient’s blood pressure levels at the time of reperfusion on short- and long-term graft and patient outcomes in SPKT recipients.

## 2. Material and Methods

### 2.1. Study Design and Study Population

The study protocol was approved by the local ethics committee of the University of Leipzig (AZ: Nr: 111-16-14032016). From a prospectively collected electronic database, we retrospectively analyzed medical data of all 109 patients who underwent SPKT at the University Hospital of Leipzig between 2000 and 2017. We especially focused on the influence of peri- and intraoperative hemodynamic parameters on long-term graft function and outcome. Patients younger than 18 years, receiving kidney transplantation alone (KTA), pancreatic retransplantation, and patients with insufficient/missing data about preoperative, perioperative, and postoperative hemodynamic parameters and outcomes were excluded from our study. The mean follow-up period of the study was 121 ± 34.4 months.

### 2.2. Outcome Analysis

Standard demographic and clinicopathological characteristics were collected and analyzed before, at the time point of, and after transplantation in the follow-up period for each patient: the pretransplantation data included recipient and donor characteristics such as age, sex, body mass index (BMI), donor causes of death, and donor’s comorbidities/clinical course (catecholamine use, creatinine value, arterial hypertension, intensive care unit length of stay (ICU-LOS)). Recipient data included a history of diabetes mellitus, time on the waiting list for organ transplantation, history and duration of pretransplantation dialysis, data of metabolic endocrine and lipid metabolism, and information on special comorbidities (e.g., presence of coronary heart disease, ejection fraction (EF) (%), peripheral arterial disease (PAD), blood pressure parameters/arterial hypertension, as well as the number of antihypertensive agents prescribed).

Peri- and postoperative data included information on operative and postoperative clinical course (operation time, blood loss, cold and warm ischemia time of the pancreas as well as kidney graft, administered amount of intraoperative infusions, number and volume of transfused blood products, acute rejection episodes (ARE), delayed renal graft function (DGF), surgical and infectious complications), immunological and immunosuppressive characteristics (human leukocyte antigen (HLA)-mismatches, cytomegalovirus (CMV)-state, induction therapy), as well as graft function and patient outcome.

Acute graft rejection was presumed when a combination of the following was observed—namely, a sudden increase in serum amylase/lipase and/or serum glucose levels in combination with a decline of serum C-peptide level, increased serum creatinine, and a reduction in urine output diuresis and abdominal pain associated with graft edema in sonographic examination. Thus, suspected acute rejection was confirmed by endoscopic biopsies of the duodenal segment of the graft whenever possible. Transcutaneous biopsies of the kidney graft were performed to confirm rejection with the omission of pancreatic biopsies due to risk-benefit evaluation. Acute cellular rejection was treated with pulsed steroids or administration of 8 mg per kg bodyweight antithymocyte globulin (ATG), together with increased baseline immunosuppression. DGF of the kidney was defined as the requirement of dialysis in the first week following transplantation [11].

### 2.3. Intraoperative Hemodynamic Monitoring and Measurement

General anesthesia was induced with intravenous propofol, fentanyl or sufentanil, and rocuronium, and maintained with isoflurane or desflurane in an oxygen-air mixture, with continuous administration of remifentanil or bolus administration of fentanyl or sufentanil and rocuronium as appropriate. Additionally, to standard monitoring, arterial pressure and central venous pressure (CVP) were continuously monitored. The arterial catheter was inserted into the radial artery, and the central venous catheter was inserted into the internal jugular vein.

According to general standards, hemodynamic indices and parameters were evaluated to guide goal-directed fluid therapy in patients undergoing major invasive surgery, particularly high-risk patients with large expected blood loss and fluid shifts, such as SPKT recipients [12,22].

Our center protocol consists of providing volume repletion and use of vasopressors, as needed, to achieve optimal and appropriate blood pressure at the time of graft reperfusion, ideally a systolic blood pressure level of >140 mmHg/MAP of >70 mmHg. In this context, the volume of fluids administered included crystalloids (mL), fresh-frozen plasma (FFP), human albumin (HA), and transfusion of erythrocyte concentrate (EC).

In the case of MAP < 70 mmHg, unresponsive to volume repletion, norepinephrine was used to maintain the targeted arterial blood pressure levels.

For assessment of hemodynamic parameters in SPKT, the following data were collected and analyzed: central venous pressure (CVP), mean arterial pressure (MAP), and heart rate (HF) at the start of incision, at the time of clamping, at the time of completion of anastomosis (reperfusion), and thereafter.

### 2.4. Surgical Techniques/Immunosuppression

As described previously, pancreas and kidney grafts were procured and transplanted following the international standards and guidelines [5,9,23,24,25]. In short, the pancreas was explanted in a no-touch technique en-bloc with the spleen and duodenum. Back-table preparation included the removal of the spleen and peripancreatic fat. Reconstruction of the superior mesenteric and the lineal artery was performed using the donor iliac Y-graft. The pancreas was transplanted into the right iliac fossa using a standard technique with an intraperitoneal location in the right iliac fossa. The Y-graft was anastomosed to the recipient’s common iliac artery using 6-0 Prolene running sutures. The portal vein was connected to the inferior vena cava of the recipient [9,23]. Exocrine drainage was carried out with a hand-sutured side-to-side duodenojejunostomy 40 cm beyond the flexure of Treitz. All kidneys were transplanted into the contralateral iliacal fossa. Vascular anastomoses were performed to the external iliac artery and vein. The ureter was implanted into the bladder according to the Lich–Gregoir technique, using a double J catheter as an intraurethral splint [26]. Splint removal was performed 3–4 weeks after successful transplantation.

The immunosuppressive protocol consisted of induction therapy, followed by triple maintenance therapy, as described previously [26,27]. Shortly, for induction therapy, antithymocyte globulin (Thymoglobulin^®^) or the interleukin-2 receptor antagonist basiliximab (Simulect^®^) was used. Maintenance therapy included calcineurin inhibitors (Cyclosporin (Sandimmun Neoral^®^) or tacrolimus (Prograf ^®^), and/or antimetabolites (Sirolimus (Rapamune^®^), mycophenolate mofetil (MMF); (Cell Cept^®^), mycophenolate sodium (Myfortic^®^), and tapered steroids (Prednisolone^®^).

### 2.5. Statistical Analysis

With regard to baseline data, continuous variables are reported as mean values with standard deviation, whereas categorical variables are presented as whole numbers and percentages (%). For analysis of baseline data, we used the appropriate statistical significance tests, including Student’s *t*-test, χ^2^, analysis of variance (ANOVA), Kruskal–Wallis and Wilcoxon–Mann–Whitney test.

To evaluate prognostic accuracy and find the appropriate cut-off values of MAP at the time of reperfusion for predicting early graft survival/failure (within the first 3 months after SPKT), a receiver operating characteristic (ROC) curve analysis for MAP and early pancreatic graft function was generated, and the area under the curve (AUC) was calculated.

According to the results, patients were divided into two groups—MAP > 91 mmHg versus MAP < 91 mmHg—for analysis of primary and secondary endpoints.

The primary endpoint of our study was long-term pancreas allograft failure following SPKT.

In this context, pancreas graft failure was defined as resumed insulin therapy, removed pancreas graft, retransplantation, or patient death. Kidney graft failure was defined as the need for dialysis, removed kidney graft, retransplantation, or patient death.

The secondary endpoint included the occurrence of renal delayed graft function, which was defined as a necessity of dialysis in the first week after transplantation or failure of creatinine to fall by 50% independently in the first week [11].

A study of potential prognostic risk factors for the secondary endpoint was carried out using logistic regression models. In the final multivariable model, potential risk factors with significant values in univariate analysis and/or known risk factors from the literature were selected using a stepwise backward selection procedure with adjustment for potential confounders. Results of the regression analysis were presented as odds ratio (OR) with 95% confidence interval (CI) and corresponding *p*-value. For the estimation of survival rates, the Kaplan–Meier method was used, and the log-rank test was applied to test statistical significance between groups.

Graft survival was calculated as the time from initial transplant to graft failure, censoring for death with a functioning graft, and grafts still functioning at the time of analysis. Patient survival was defined as the time from transplant to patient death, censoring for patients still alive at the time of analysis. If a recipient was alive or lost to follow-up at the time of the last contact, then survival time was censored at the time of the last contact.

Cox proportional hazard regression models were used to calculate hazard ratios (HR), with 95% confidence intervals (CI) for the primary endpoints. Variables to be entered into the multiple logistic regression analysis were chosen on the basis of the results of univariate analysis. A *p* value < 0.05 was considered statistically significant.

All statistical analyses were performed by using SPSS software (SPSS Inc., Chicago, IL, USA, version 21.0).

## 3. Results

### 3.1. Baseline Characteristics

Between January 2000 and July 2017, a total of 105 patients following SPKT at the University Hospital of Leipzig could be included in the study.

At ROC curve analysis, the optimal cut-off point for MAP at reperfusion to predict early pancreas graft failure was 91 mmHg.

The area under the curve was (AUC) was 0.63 (95%CI: 0.60–0.66), with a sensitivity of 39.9% and a specificity of 87.7% (*p* = 0.017).

Demographic and clinicopathologic baseline characteristics of the two groups according to their MAP values are compared and shown in Table 1.

Patients with >91 mmHg at reperfusion had significantly shorter durations of the intensive care unit (*p* = 0.017) an ad significantly higher EF rates (*p* = 0.04). No other significant baseline characteristics could be observed between both groups.

### 3.2. Intraoperative Outcomes and Measurements

Table 2 shows the intraoperative outcome parameters of the study group.

In the MAP > 91 mmHg group, the total intraoperative urine output was significantly higher than that in the MAP < 91 mmHg group (MAP > 91 mmHg versus MAP < 91 mmHg: 195 ± 45 mL versus 116 ± 50 mL; *p* = 0.04). Further, the amount of intraoperative administered fluids were higher in the MAP < 91 mmHg group (MAP < 91 mmHg 4436 ± 1447 mL versus MAP > 91 mmHg 3945 ± 1643 mL; *p* = 0.110) but showed no significant difference.

There were no differences in operating time (MAP > 91 versus MAP < 91: 372 ± 81 min versus 391 ± 115 min; *p* = 0.352), warm ischemic time pancreas (MAP > 91 versus MAP < 91: 37.3 ± 5.8 min versus 39.8 ± 19.2 min; *p* = 0.456) and kidney (MAP > 91 mmHg versus MAP < 91 mmHg: 33.2 ± 2.5 min versus 35.5 ± 6.8 min; *p* = 0.348), cold ischemic time of pancreas (MAP > 91 mmHg versus MAP < 91 mmHg: 10.8 ± 2.1 h versus 11.2 ± 2.7 h; *p* = 0.123) and kidney (MAP > 91 mmHg versus MAP < 91 mmHg: 11.2 ± 1.8 h versus 11.8 ± 2.3 h; *p* = 0.356), blood loss (MAP > 91 mmHg versus MAP < 91 mmHg, 1051 ± 960 mL versus 812 ± 563 mL; *p* = 0.317), transfusion of erythrocyte concentrates (MAP > 91 mmHg versus MAP < 91 mmHg: 390 ± 70.1 versus 300 ± 78.6; *p* = 0.381), or fresh-frozen plasma (MAP > 91 mmHg versus MAP < 91 mmHg: 230 ± 54.8 mL versus 122 ± 50.1 mL; *p* = 0.167).

**Figure 1:**
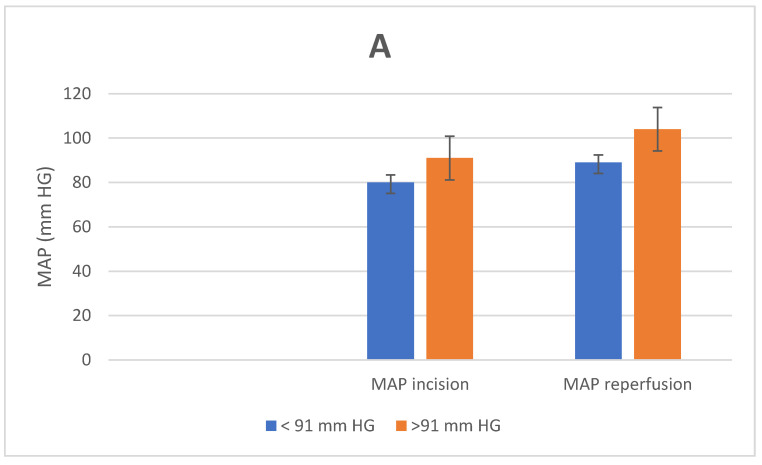

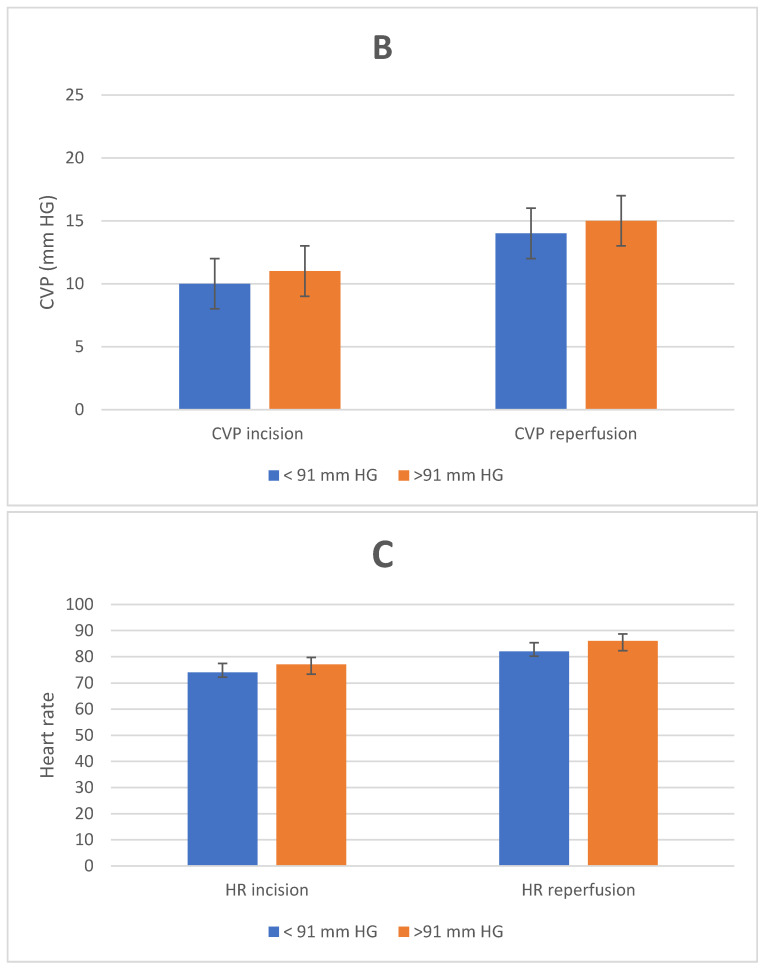
Intraoperative hemodynamic measurements: (**A**) mean arterial pressure (MAP); (**B**) central venous pressure (CVP); (**C**) heart rate (HR).

Figure 1 shows the hemodynamic measurements of the two different groups at the time of incision and at the time of reperfusion.

The hemodynamic parameters were not statistically different at the time of incision. The MAP at the time of reperfusion was 104 ± 9.8 in the MAP > 91 mmHg group and 89 ± 3.4 in the MAP < 91 mmHg group (*p* = 0.01). No differences were seen in CVP (MAP > 91 mmHg versus MAP < 91 mmHg: 15 ± 3.5 mmHg versus 14 ± 4 mmHg; *p* = 0.589) and heart rate (HF) (MAP > 91 mmHg versus MAP < 91 mmHg: 77 ± 2.7 bpm versus 74 ± 3.4 bpm; *p* = 0.239) between both groups.

### 3.3. Postoperative Outcome

Postoperative outcome parameters of the recipients according to their MAP values are illustrated in Table 3. There was a higher incidence of DGF of the kidney (MAP < 91 mmHg versus MAP > 91 mmHg: 23% versus 9%; *p* = 0.03), higher rates of pancreatitis/intraabdominal pancreatic infectious fluid formations (MAP < 91 mmHg versus MAP > 91 mmHg: 21% versus 7%; *p* = 0.03), and higher rates of vascular thrombosis of the pancreas (MAP < 91 mmHg versus MAP > 91 mmHg: 14% versus 3%; *p* = 0.03) in the MAP < 91 mmHg group.

There were no significant differences with regard to acute rejection episodes (*p* = 0.481), rate of reoperations (*p* = 0.858), bleeding episodes (*p* = 0.756), and CMV infections (*p* = 0.974) between both groups.

### 3.4. Metabolic Outcome

In terms of the metabolic outcome, recipients of the MAP > 91 mmHg group showed significant better HbA1c- values at 1 year (MAP > 91 mmHg versus MAP < 91 mmHg: 5.5 ± 0.7 versus 6.1 ± 1.2; *p* = 0.032), 3 years (MAP > 91 mmHg versus MAP < 91 mmHg: 5.4 ± 0.9 versus 5.9 ± 1.3; *p* = 0.013), and 5 years (MAP > 91 mmHg versus MAP < 91 mmHg: 5.6 ± 1.8 versus 6.5 ± 1.8; *p* = 0.047) after transplantation.

There were no significant differences in terms of C-peptide values, creatinine and urea values, LDL/HDL ratios, and systolic blood pressure values at 6, 12, 36, and 60 months after transplantation (Table 4).

### 3.5. Primary Endpoint

**Figure 2:**
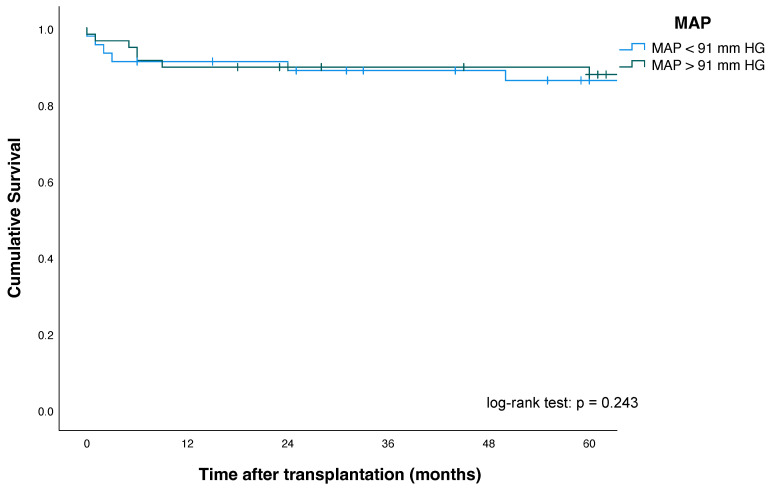
Patient survival according to mean arterial pressure (MAP) after simultaneous pancreas-kidney transplantation.

**Figure 3:**
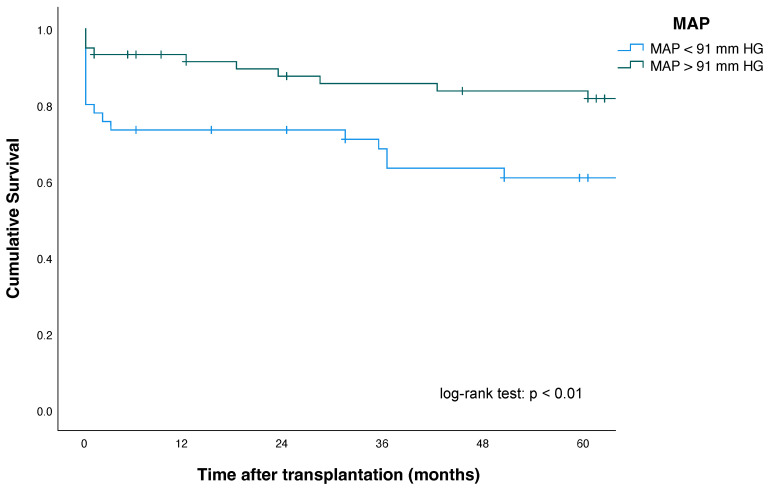
Pancreas graft survival according to mean arterial pressure (MAP) after simultaneous pancreas–kidney transplantation.

**Figure 4:**
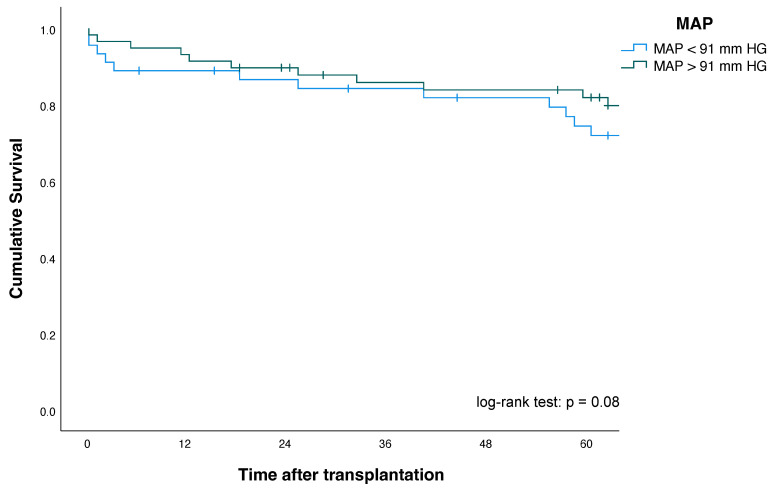
Kidney graft survival according to mean arterial pressure (MAP) after simultaneous pancreas–kidney transplantation.

Kaplan–Meier plots for patients, as well as pancreas and kidney graft survival according to their MAP status, are shown in Figure 2, Figure 3 and Figure 4.

Five-year pancreas graft survival (63% vs. 82%; *p* = 0.014) and three-month pancreas graft survival (76% vs. 91%; *p* = 0.02)—as defined by the ROC analysis—were significantly lower in patients with MAP < 91 mmHg at the time point of reperfusion, compared with patients with MAP > 91 mmHg.

One-, three- and five-year patient survival and kidney graft survival among MAP < 91 mmHg and MAP > 91 mmHg groups showed no significant differences. For improved risk assessment, the ROC analysis defining factor (MAP >/< 91 mmHg) was intentionally included as a single univariate risk factor in the explorative multivariate Cox regression analysis to assess its validity and robustness (see also Section 3.6). Here, multivariate Cox regression analysis revealed that a MAP < 91 mm HG (HR, 2.26 (95% CI: 1.06–4.8), *p* = 0.01) was an independent and significant predictor and risk factor of increased pancreas allograft failure in our study population (Table 5).

Furthermore, donor age and BMI, recipient BMI, donor cause of death, >12 h cold ischemia time (CIT) of the pancreas, as well as DGF, were also revealed as independent and significant predictors for pancreas graft failure in our study analysis.

No significant differences could be observed between MAP < 91 mmHg and kidney allograft failure (HR 1.90 (95% CI 0.93–3.84), *p* = 0.08) and patient death (HR, 1.31 (95% CI, 0.52–3.31), *p* = 0.568).

### 3.6. Secondary Endpoint

With regard to secondary endpoints, several prognostic factors in univariate analysis were identified for DGF following SPKT: MAP < 91 mmHg (HR 3.63, 95% CI: 1.18–11.22; *p* = 0.02), donor age (HR 1.23, 95% CI: 1.12–1.34; *p* = 0.001), and duration of pretransplant dialysis (HR 1.03, 95% CI: 1.01–1.05; *p* = 0.002). Multivariate logistic regression analysis revealed MAP < 91 mmHg, donor age, and duration of pretransplant dialysis as independent risk factors for DGF (Table 6).

## 4. Discussion

Successful SPKT is mainly dependent on the optimization of several clinical and paraclinical parameters [5,10,28]. To the best of our knowledge, this is the first study investigating the effect of MAP at reperfusion on graft function and postoperative outcome, as well as long-term graft survival, in a larger cohort of SPKT recipients.

Adequate organ perfusion, combined with appropriate blood pressure and volume repletion during organ transplantation, is essential to avoid hypoxia, the leading cause of organ dysfunction and damage [12,16,29]. The hemodynamic status of the recipient during the transplantation, the time following the transplant, particularly with completion of the anastomosis, and the time point of reperfusion can affect graft perfusion and showed significant influence on short- and long-term outcome and graft function [14,15,16,19,21]. Today, it is widely acknowledged and supported by various studies that such perioperative hemodynamic factors can influence the immediate and long-term graft function and outcome [12,13,14,15,16,19,21,29,30,31]. Consequently, it is of importance to define those hemodynamic monitoring parameters in detail, which correlate with the development of early postoperative complications (e.g., DGF) and consecutively influence long-term graft function and outcome.

Although some retrospective data concerning perioperative hemodynamic parameters exist in KTA recipients, the optimal blood pressure level and perfusion pressure gradient in SPKT recipients, which are needed for an adequate flow in individualized patient management, have not been determined. As previously described, a MAP lower than 70 mmHg, as well as low volume repletion (CVP < 8 mmHg) at the time of reperfusion, was frequently associated with DGF in renal transplant recipients [13,14,15,19]. On the other hand, an intraoperative MAP > 95 mmHg and a moderate volume repletion (CVP > 12 mmHg) at the time of reperfusion was associated with immediate graft function and better long-term graft survival in previous studies [14,21]. These results are in accordance with our findings in multivariate analysis of SPKT recipients that a MAP < 91 mmHg at reperfusion lead to renal DGF, which is associated with worse early graft function and inferior long-term pancreas graft survival. However, the evaluation of the phenomena DGF is notoriously complicated due to several contributing factors that are partly related to the pathophysiological characteristics of the recipient, donor, or surgeon, as well as hemodynamic/immunological processes and compensation of the comorbidities, even in the early postoperative phase [8,11]. Nevertheless, the low incidence rates of DGF in the MAP > 91 mmHg group suggest that immediate graft function is key to reducing the incidence of early postoperative complications and enhancing long-term survival, particularly in high-risk patients.

Concerning the observed differences in baseline data, the MAP < 91 mmHg group had a lower EF before SPKT than patients with MAP > 91 mmHg at reperfusion. Of interest is the finding that the MAP < 91 mmHg group also showed a trend toward longer exposure to hemodialysis, as well as a trend toward a longer waiting time before transplantation. These findings may to some extent contribute to difficulties in achieving an adequate MAP during reperfusion due to decreased cardiac output and aggravated vasoplegia in the MAP < 91 mmHg group. However, despite some intraoperative efforts achieving an appropriate MAP at reperfusion, there were no significant differences between both groups in terms of CVP and administration of total fluid volume or catecholamine at the time point of reperfusion. Concerning major perioperative/surgical-related complications, the differences in incidence rates between both groups further highlighted the remarkable role of optimum blood pressure levels at the time of reperfusion. Though there were no differences in glomerular filtration rate (GFR) and metabolic factors post-transplant, securing adequate blood pressure and organ perfusion in the early postoperative phase seems to be vitally important for early recovery from DGF and to optimize adequate graft function and long-term survival.

Optimum perioperative fluid administration with adequate intravascular volume maintenance is essential to improve outcomes in critically ill patients, prevent acute organ failure or dysfunction, and improve immediate graft function after KTA and SPKT [19,21,31,32,33,34,35]. Determination of the perfect amount of fluid therapy is highly challenging in critically ill patients, as well as in the perioperative phase during operations with a high fluid turnover. Particularly in KTA and SPKT patients with a history of chronic kidney disease and potentially preoperative dependence on hemodialysis, assessment of adequate intravascular volume status is highly challenging due to common comorbidities such as advanced age, reduced left ventricular function, vascular calcification, and decreased vascular reactivity because of autonomic neuropathy, also referred to as “dialysis-related hypotension” [36,37,38]. In these patients, during states of reduced preload, adequate cardiac filling volume and consequent stroke volume will mostly depend on left ventricular cardiac diastolic function, which is often impaired after long-term dialysis [39]. Additionally, abnormal calcium and phosphate metabolism and secondary hyperparathyroidism worsen the progression of arteriosclerosis due to vascular calcification spreading from the media to the intima, contributing to a decreased responsiveness to vasoconstriction [38]. These factors may deteriorate compensatory autonomic reflexes with peripheral vasoconstriction and eventually lead to paradoxical hypotension [36,40,41].

In this context, the use of extended hemodynamic monitoring—especially in high-risk patients such as SPKT recipients—may be a helpful tool to assess intravascular volume status and fluid responsiveness enabling an individualized perioperative volume and organ perfusion approach to reduce morbidity and mortality during SPKT and improve graft function and outcome [13,19,32]. CVP-guided volume infusion has been the most popular and traditional approach of guiding and monitoring fluid therapy in KTA during the last several years [19,32]. However, findings of previous studies on the assessment of intravascular volume, optimization of cardiac output, and renal blood flow with regard to graft function and outcome have indicated that conventional monitoring parameters such as CVP to guide fluid management in KTA provides insufficient and unreliable data with cut-off values of CVP ranging between 5 and 15 mmHg at the time of reperfusion [13,14,19,32,42].

In our current study, CVP (14 ± 4 mmHg in the MAP < 91 mmHg group versus 15 ± 4.5 in the MAP > 91 mmHg group, *p* = 0.589), as well as administered total volume (4436 ± 1447 mL in the MAP < 91 mmHg group versus 3945 ± 1643 mL in the MAP > 91 mmHg group, *p* = 0.110), were comparable between both groups at reperfusion. Thus, volume management was considered to have been performed appropriately and without significant differences between both groups.

However, optimal perioperative fluid administration in KTA and SPKT remains a black box, reinforced by the fact that fluid requirements are highly variable among different patients and different surgical procedures and the assessment of an individual patient’s intravascular volume remains challenging [19]. Recent medical advances in understanding the hemodynamic properties of the vascular system, together with the availability of new technologies to precisely measure the effect of intravenous fluids on cardiac output, have changed the scope of diagnostic approaches.

Currently, several technologies and equipment for assessing dynamic preload and cardiac output variables of fluid response such as pulse contour analyses, arterial waveform derived variables (i.e., systolic pressure variation (SPV), pulse pressure variation (PPV), stroke volume variation (SVV)), thoracic electrical bioimpedance, lithium dilution or partial CO_2_ rebreathing measurements, and intraoperative transesophageal Doppler (TED) were introduced in specific patient populations for optimal fluid management [19,43].

However, prospective comparative clinical studies are lacking to check the reliability of these new techniques to guide the optimal fluid therapy in surgical cases during the clinical routine setting.

To date, there are only sparse data on the use and effect of vaso-inotropic agents on postoperative graft function in renal transplant recipients, and no data on SPKT exist [13,17]. Although the amount of cumulative administered catecholamine is higher in the MAP < 91 mmHg group and consequently associated with more DGF, this parameter was not detected as an independent risk factor for DGF in the multivariate regression analysis. It is not surprising that patients with arterial hypotension need more volume and/or vasoactive agents at reperfusion so that correlations may be present; however, they are not necessarily conclusive for DGF.

Several limitations of our study are important to illustrate.

Firstly, the low number of patients in each group and the retrospective non-randomized design need to be discussed. Thus, there is a possibility of insufficient power to detect small effects and although adjustments were made for some confounders, residual confounding effects could not be ruled out completely. Moreover, as the results of our ROC analysis showed that the AUC of MAP at reperfusion is only just above over 0.6 and thus represents a weak correlation, we included this parameter in an explorative multivariate Cox regression analysis to check its robustness and validity. In addition, we only observed that the differences in MAP at the time of reperfusion are statistically correlated with long-term graft function; therefore, it is completely unclear if an increase in MAP results in better graft function, and if so, which measures to increase MAP may translate into better graft function. Therefore, to test the hypothesis that therapeutically increased MAP might result in better graft function, prospective studies in this interesting field are mandatory.

Secondly, the long investigation period of our study and slight changes in anesthesiologic management may have affected therapeutical decisions and restricted data evaluation. Thus, there may be undetected factors such as the ability to maintain high blood pressure and the effectiveness of vaso-active inotropic agents in individual recipients. However, perioperative hemodynamic management and volume administration did not change relevantly during the study period.

Third, a mismatch between recipients and donor arterial blood pressure levels prior to terminal events (often unknown) may also affect intraparenchymal hemodynamics in a way undetectable by standard clinical perfusion measurements. Our study showed no significant correlation between donor hypotension episodes and incidence of DGF and reduced long-term outcome; however, these confounding effects could not be completely ruled out.

## 5. Conclusions

Appropriate blood pressure levels and optimum blood flow in the peri- and postoperative phases are crucial to early graft function and long-term graft outcome. A MAP > 91 mmHg is a strong independent parameter for an increased pancreas long-term survival, as well as enhanced graft function and outcome. Further prospective and controlled studies with larger patient populations are necessary to evaluate long-term prognosis in SPKT recipients to verify our results and lead to a better understanding of the use of dynamic analyses of flow parameters for optimal fluid management in these specific patients who often have impaired cardiovascular physiology and reduced hemodynamic autoregulation due to end-stage kidney disease and diabetes mellitus.

## Figures and Tables

**Table 1:** Baseline perioperative transplant characteristics of recipient and donor.

Variables			<91 mm HG (*n* = 47)	>91 mm HG (*n* = 58)	*p*-Value
**Donor**					
	Age, years		28.5 ± 12.1	24.7 ± 11.4	0.605
	Gender, male/female		18(38)/29(62)	23(40)/35(60)	0.887
	BMI, kg/m^2^		21.5 ± 3.4	22.4 ± 3.5	0.174
	katecholamine use		36 (76)	39 (67)	0.291
	Intensiv care unit, days		3.6 ± 3.9	2.1 ± 2.2	0.017
	Creatinine (ummol/L)		81.8 ± 10.9	84.8 ± 7.3	0.817
	Hypertension, *n* (%)		5 (11)	6 (10)	0.964
**Recipient**					
	Age, years		42.1 ± 9.1	43.2 ± 9.2	0.537
	Gender, male/female		23 (49)/24 (51)	25 (43)/33 (57)	0.551
	BMI, kg/m^2^		24.9 ± 4.2	24.9 ± 4.4	0.997
	HbA1c, (%)		8.0 ± 1.5	7.8 ± 1.9	0.218
	Duration of Diabetes, years		25.7 ± 8.8	27.8 ± 1.9	0.431
Comorbidities					
	Cardiovascular disease, *n* (%)		13 (28)	18 (31)	0.706
	Peripheral arterial disease, *n* (%)		6 (13)	11 (19)	0.391
	Hypertension, *n* (%)		38 (81)	46 (79)	0.843
	Number antihypertensive medications		2.9 ± 1.7	2.4 ± 1.6	0.143
	Peroperative Hb (ummol/L)		7.5 ± 1.0	7.4 ± 1.4	0.647
	Previous dialysis, *n* (%)		36 (76)	47 (81)	0.578
	Duration of dialysis, months		41.9 ± 3.7	28.6 ± 3.1	0.080
	Waiting time, months		11.7 ± 13.4	7.6 ± 9.9	0.084
	Ejection fraction (%)		59.6 ± 1.5	64.2 ± 1.6	0.040
**Transplant characteristics**					
	CMV D+/R−		7 (15)	13 (22)	0.342
	HLA mismatches > 2/6		35 (74)	40 (69)	0.534
	Immunosuppression				
		Induction therapy (ATG/SRL/None)	30/12/5 (65/26/9)	39/13/6 (67/22/11)	0.925
		CNI, FK506/CsA/	41/6 (87/13)	55/3 (95/5)	0.166
		AP drug, MMF/SRL/none	38/7/2 (81/15/4)	49/8/1 (85/14/1)	0.723

**Table 2:** Intraoperative outcome and measurements.

Variables		<91 mm HG (*n* = 47)	>91 mm HG (*n* = 58)	*p*-Value
Cold ischemia time, hours				
	Pancreas	11.2 ± 2.7	10.8 ± 2.1	0.123
	Kidney	11.8 ± 2.3	11.2 ± 1.8	0.356
Warm ischemia time, minutes				
	Pancreas	39.8 ± 19.2	37.3 ± 5.8	0.456
	Kidney	35.5 ± 6.8	33.2 ± 2.5	0.348
Operating time, hours		391 ± 115	372 ± 81	0.352
Intravenous infusions, mL				
	Total	4436 ± 1447	3945 ± 1643	0.110
Total transfusion				
	Red blood cell	300 ± 78.6	390 ± 70.1	0.382
	Fresh-frozen plasma (FFP)	122 ± 50.1	230 ± 54.8	0.167
	Catecholamine (mg)	134 ± 124	111 ± 93	0.224
Postreperfusion urin output, 1 h, (mL)		116 ± 50	195 ± 45	0.040
Blood loss (mL)		812 ± 563	1051 ± 960.3	0.317
Catecholamine use		39 (83)	39 (67)	0.067

**Table 3:** Postoperative complications after simultaneous pancreas–kidney transplantation.

Variables		<91 mm HG (*n* = 47)	>91 mm HG (*n* = 58)	*p*-Value
Delayed graft function (%)				
	Kidney	11 (23%)	5 (9%)	0.030
Combined acute rejection episodes (%)		7 (15%)	6 (10%)	0.481
Pancreatitis/intraabominal abscess		10 (21%)	4 (7%)	0.030
Vascular thrombosis pancreas (%)		7 (14%)	2 (3%)	0.030
Reoperation		17 (36%)	20 (34%)	0.858
Bleeding		4 (8%)	4 (7%)	0.756
CMV Infection		9 (19%)	11 (19%)	0.974

**Table 4:** Metabolic outcome after simultaneous pancreas–kidney transplantation.

Variables	Time after SPKT											
	6 Months			1 Year			3 Year			5 Years		
	<91 mm HG	>91 mm HG	*p*-Value	<91 mm HG	>91 mm HG	*p*-Value	<91 mm HG	>91 mm HG	*p*-Value	<91 mm HG	>91 mm HG	*p*-Value
C-peptide, ng/mL	2.3 ± 1.9	2.9 ± 1.7	0.289	2.0 ± 0.4	1.5 ± 0.6	0.342	0.8 ± 0.5	1.1 ± 0.8	0.146	1.1 ± 0.6	0.9 ± 0.4	0.514
HbA1c, %	5.8 ± 0.15	5.6 ± 0.2	0.178	6.1 ± 1.2	5.5 ± 0.7	0.032	5.9 ± 1.3	5.4 ± 0.9	0.013	6.5 ± 1.8	5.6 ± 0.6	0.047
Creatine, mmol/L	132 ± 11	124 ± 8	0.591	135 ± 12	117 ± 7	0.318	135 ± 12	116 ± 11	0.214	150 ± 1.6	123 ± 11.2	0.220
Urea, mmol/L	8.8 ± 0.5	7.9 ± 0.6	0.239	10.1 ± 1.5	8.4 ± 0.8	0.210	9.5 ± 0.8	8.3 ± 0.6	0.387	11.3 ±1.8	9.2 ± 0.9	0.789
LDL/HDL ratio	2.1 ± 1.1	1.9 ± 0.8	0.207	2.0 ± 0.4	1.7 ± 0.5	0.678	1.9 ± 0.9	1.6 ± 0.5	0.210	2.1 ± 1.3	1.9 ± 0.9	0.748
Systolic blood pressure (mm HG)	127 ± 3	125 ± 2	0.603	120 ± 5	124 ± 3	0.263	126 ± 4	128 ± 3	0.419	134 ± 5	129 ± 3	0.197

**Table 5:** Multivariate Cox regression analysis of predictors for pancreas allograft failure after simultaneous pancreas–kidney transplantation.

Variables			Univariate Analysis			Multivariate Analysis		
			HR	95 CI	*p*-Value	HR	95 CI	*p*-Value
**Donor**								
	Age		1.04	1.01–1.07	0.006	1.03	1.01–1.07	0.04
	Gender (male versus female)		1.87	0.93–3.74	0.075			
	Body mass index (BMI) (kg/m^2^)		1.15	1.01–1.24	0.027	1.16	1.03–1.30	0.01
	Cause of death (Non-trauma vs. trauma)		8.1	1.8–92.6	0.01	6.9	1.57–29.8	0.019
	Serum creatinine (ummol/L)		1.01	0.99–1.07	0.587			
	Katecholamine use		0.95	0.43–2.08	0.901			
**Recipient**								
	Age		1.046	1.00–1.09	0.03	1.032	0.98–1.08	0.157
	Gender (male vs. female)		0.54	0.29–1.02	0.059			
	Body mass index (BMI) (kg/m^2^)		1.09	1.02–1.17	0.015	1.17	1.02–1.22	0.014
	Duration of pretransplant diaylsis (months)		0.99	0.98–1.01	0.826			
	Duration of diabetes, years		1.01	0.97–1.05	0.512			
**Procedural risk factors**								
	Total volume intraoperative fluids (mL)		1.01	0.98–1.03)	0.665			
	Mean arterial Pressure < 91 mm HG *		2.86	1.49–5.47	0.002	2.26	1.06–4.8	0.01
	Blood transfusion		1.75	0.88–3.45	0.105			
**Transplant**								
	Warm ischemia time, min							
		Pancreas	1.01	0.23–5.2	0.967			
		Kidney	1.08	0.21–6.42	0.89			
	CIT, hours							
		Pancreas, >12 h	5.82	1.34–25.2	0.018	4.48	1.96–10.2	0.001
		Kidney, >12 h	1.47	0.43–5.07	0.53			
	Delayed graft function kidney		2.42	1.07–5.46	0.029	2.44	1.07–5.53	0.033
	Acute rejection episodes		1.75	0.88–3.46	0.105			
	PRA > 10%		1.25	0.67–2.37	0.481			
	HLA-mismatch		1.01	0.82–1.19	0.951			
**Immunosuppression**								
	Induction therapy							
		None	Ref.		0.342			
		ATG	0.58	0.16–2.08	0.408			
		IL-2 RA	0.44	0.13–1.51	0.19			
	CNI (Tac versus CSA)		0.42	0.12–1.43	0.164			
	AP drug							
		None	Ref.		0.292			
		MMF	0.4	0.54–2.98	0.371			
		SRL	0.18	0.01–1.78	0.100			

* Group-defining factor.

**Table 6:** Multivariate logistic regression analysis of predictors for delayed kidney graft function after simultaneous pancreas–kidney transplantation.

Variables			Delayed Graft Function					
			Univariate Analysis			Multivariate Analysis		
			OR	95 CI	*p*-Value	OR	95 CI	*p*-Value
**Donor**								
	Age (years)		1.23	1.12–1.34	0.001	1.18	1.08–1.29	0.001
	Gender (male vs. female)		0.27	0.07–1.04	0.051			
	Body mass index (BMI) (kg/m^2^)		1.09	0.92–1.30	0.271			
	Death due to CVA		1.50	0.75–2.87	0.198			
	Serum creatinine (ummol/L)		1.05	0.99–1.01	0.166			
	Katecholamine use		1.30	0.34–5.06	0.704			
**Recipient**								
	Age (years)		1.02	0.96–1.08	0.439			
	Gender (male vs. female)		1.67	0.56–4.92	0.349			
	Body mass index (BMI)		1.05	0.93–1.18	0.453			
	Duration of pretransplant dialysis (months)		1.03	1.01–1.05	0.002	1.03	1.03–1.06	0.031
	Duration of diabetes (years)		0.99	0.94–1.06	0.975			
**Procedural risk factors**								
	Blood transfusion		1.03	0.36–2.86	0.967			
	Mean arterial Pressure < 91 mm HG *		3.63	1.18–11.22	0.020	3.49	1.12–10.84	0.035
	Total volume intraoperative fluids (mL)		0.98	0.97–1.04	0.110			
**Transplant**								
	HLA mismatch > 2		1.11	0.80–1.56	0.398			
	PRA > 10%		1.89	0.50–6.24	0.291			
	Cold ischemia time							
		Pancreas	1.02	0.99–1.05	0.188			
		Kidney	1.01	0.08–1.04	0.530			
	Warm ischemia time							
		Pancreas	0.98	0.94–1.02	0.343			
		Kidney	1.04	0.97–1.04	0.814			
		Induction therapy (ATG vs. Simulect)	1.8	0.6–5.36	0.456			

* Group-defining factor.

## Data Availability

Our database contains highly sensitive data that may reveal clinical and personnel information about our patients and lead to their identification. Therefore, according to organizational restrictions and regulations, these data cannot be made publicly available. However, the datasets used and/or analyzed in the current study are available from the corresponding author upon reasonable request.

## Abbreviations

ANOVA	Analysis of variance
ARE	Acute rejection episode
ATG	Antithymoctye globulin
AUC	Area under the curve
BMI	Body mass index
CI	Confidence interval
CIT	Cold ischemia time
CMV	Cytomegalovirus
CNI	Calcineurin inhibitor
CVP	Central venous pressure
DGF	Delayed graft function
EC	Erythrocyte concentrate
EF	Ejection fraction
ESKD	End-stage kidney disease
FFP	Fresh-frozen plasma
GFR	Glomerular filtration rate
HA	Human albumin
HF	Heart rate
HLA	Human leukocyte antigen
HR	Hazard ratio
ICU-LOS	Intensive care unit length of stay
IDDM1	Insulin-dependent diabetes mellitus type 1
KTA	Kidney transplantation alone
MAP	Mean arterial pressure
MMF	Mycophenolate mofetil
OR	Odds ratio
PAD	Peripheral arterial disease
PPV	pulse pressure variation
QoL	Quality of life
ROC	Receiver operating characteristic
SPKT	Simultaneous pancreas–kidney transplantation
SPV	Systolic pressure variation
SRL	Sirolimus
SVV	Stroke volume variation
TED	Transesophageal doppler
